# Incidence and risk factors of postoperative pulmonary complications following total hip arthroplasty revision: a retrospective Nationwide Inpatient Sample database study

**DOI:** 10.1186/s13018-024-04836-3

**Published:** 2024-06-14

**Authors:** Liping Huang, Xinlin Huang, Junhao Lin, Qinfeng Yang, Hailun Zhu

**Affiliations:** 1 School of Health, Dongguan Polytechnic, Dongguan, Guangdong 523000 China; 2 The Second School of Clinical Medicine, Southern Medical University, Guangzhou, Guangdong 510515 China; 3 School of Traditional Chinese Medicine, Southern Medical University, Guangzhou, Guangdong 510515 China; 4 Division of Orthopaedic Surgery, Department of Orthopaedics, Nanfang Hospital, Southern Medical University, Guangzhou, Guangdong 510515 China; 5 Department of Orthopedics, Shenzhen Hospital, Southern Medical University, Shenzhen, Guangdong 518100 China

**Keywords:** Total hip arthroplasty revision, Postoperative pulmonary complications, Specific pulmonary complications, Nationwide inpatient sample

## Abstract

**Background:**

Postoperative pulmonary complications (PPCs) are among the most severe complications following total hip arthroplasty revision (THAR), imposing significant burdens on individuals and society. This study examined the prevalence and risk factors of PPCs following THAR using the NIS database, identifying specific pulmonary complications (SPCs) and their associated risks, including pneumonia, acute respiratory failure (ARF), and pulmonary embolism (PE).

**Methods:**

The National Inpatient Sample (NIS) database was used for this cross-sectional study. The analysis included patients undergoing THAR based on NIS from 2010 to 2019. Available data include demographic data, diagnostic and procedure codes, total charges, length of stay (LOS), hospital information, insurance information, and discharges.

**Results:**

From the NIS database, a total of 112,735 THAR patients in total were extracted. After THAR surgery, there was a 2.62% overall incidence of PPCs. Patients with PPCs after THAR demonstrated increased LOS, total charges, usage of Medicare, and in-hospital mortality. The following variables have been determined as potential risk factors for PPCs: advanced age, pulmonary circulation disorders, fluid and electrolyte disorders, weight loss, congestive heart failure, metastatic cancer, other neurological disorders (encephalopathy, cerebral edema, multiple sclerosis etc.), coagulopathy, paralysis, chronic pulmonary disease, renal failure, acute heart failure, deep vein thrombosis, acute myocardial infarction, peripheral vascular disease, stroke, continuous trauma ventilation, cardiac arrest, blood transfusion, dislocation of joint, and hemorrhage.

**Conclusions:**

Our study revealed a 2.62% incidence of PPCs, with pneumonia, ARF, and PE accounting for 1.24%, 1.31%, and 0.41%, respectively. A multitude of risk factors for PPCs were identified, underscoring the importance of preoperative optimization to mitigate PPCs and enhance postoperative outcomes.

**Supplementary Information:**

The online version contains supplementary material available at 10.1186/s13018-024-04836-3.

## Introduction

Among patients suffering from acute hip disease or damage, total hip arthroplasty (THA) has been shown to be a promising and beneficial operation for reducing pain, ameliorating joint deformity, and improving the patients’ quality of life [1]. Currently, about 400,000 THAs are performed each year in the USA. In addition, with the development of aging population, the demand for THA is increasing significantly, and the number is expected to increase up to 572,000 by 2030 [2].

However, after THA surgery, failure and the need for revision remain a non-negligible problem. As the number of THAs grows, total hip arthroplasty revision (THAR), a procedure that may occur due to prosthetic-related complications like joint stiffness, periprosthetic fracture, periprosthetic joint dislocation, and mechanical unraveling, has been increasingly performed around the world [1]. Unfortunately, with the increasing frequency of THARs, complications are more prevalent [3].

One of the typical complications after THAR is postoperative pulmonary complications (PPCs). Previous studies have revealed the occurrence of PPCs can impose a significantly heavy burden on medical resources and patients due to prolonged hospitalization stays, increased medical costs, and higher mortality rates [4–6]. To mitigate the substantial burden on medical resources and patients associated with PPCs, the development of a clinical PPCs risk model is imperative [7]. However, until now, there is no study about the incidence and risk factors of the PPCs following THAR. Consequently, this aim of our study was to investigate the prevalence and identify risk factors associated with PPCs after THAR, utilizing information from the NIS database. In addition, we further divided PPCs into specific pulmonary complications (SPCs) and independent and common risk factors associated with SPCs were identified. SPCs encompassed serious and potentially life-threatening PPCs, including pneumonia, acute respiratory failure (ARF), and pulmonary embolism (PE). The assessment in our study included evaluating morbidity, in-hospital mortality, the Charlson comorbidity index (CCI), patient demographics, payer type, total cost, medical and surgical perioperative complications, length of stay (LOS), as well as risk factors for PPCs following THAR.

## Materials and methods

### Data source

The Nationwide Inpatient Sample (NIS) database, a component of the Agency for Healthcare Research and Quality’s Healthcare Cost and Utilization Program, served as the basis for this cross-sectional study. As the most substantial comprehensive database of hospitalized patients in US NIS gathers a stratified sample from over 1,000 institutions, encompassing roughly 20% of all hospitals in the country annually [8]. The available data includes demographics, length of stay (LOS), diagnosis and procedure codes, total charges, hospital information, insurance information, and discharge position. This exploratory investigation utilized unidentified publicly accessible data, which means it is considered an exemption regarding the ethical committee statement.

### Data collection

The data used in this study was obtained from the NIS database, covering the period from 2010 to 2019. The identification of patients who had THAR as a main surgery between the years 2010 and 2019 was accomplished via the use of International Classification of Diseases, 9th Revision, Clinical Modification (ICD-9-CM) and International Classification of Diseases, 10th Revision, Clinical Modification (ICD-10-CM) procedural codes (ICD-9-CM: 00.70/00.71/00.72/00.73/80.05/81.53; ICD-10-CM: 0SP-X/0SW-X). According to previous studies [9, 10], major PPCs were defined as pneumonia (ICD-9-CM: 480.0–486; ICD-10-CM: J12.0–J17), ARF (ICD-9-CM: 518.81; ICD-10-CM: J96.01/J96.02), PE (ICD-9-CM: 415.11/415.12//415.13/415.19; ICD-10-CM: I26.02/I26.09/J96.92/J96.93/J96.94/J96.99).

**Fig. 1:**
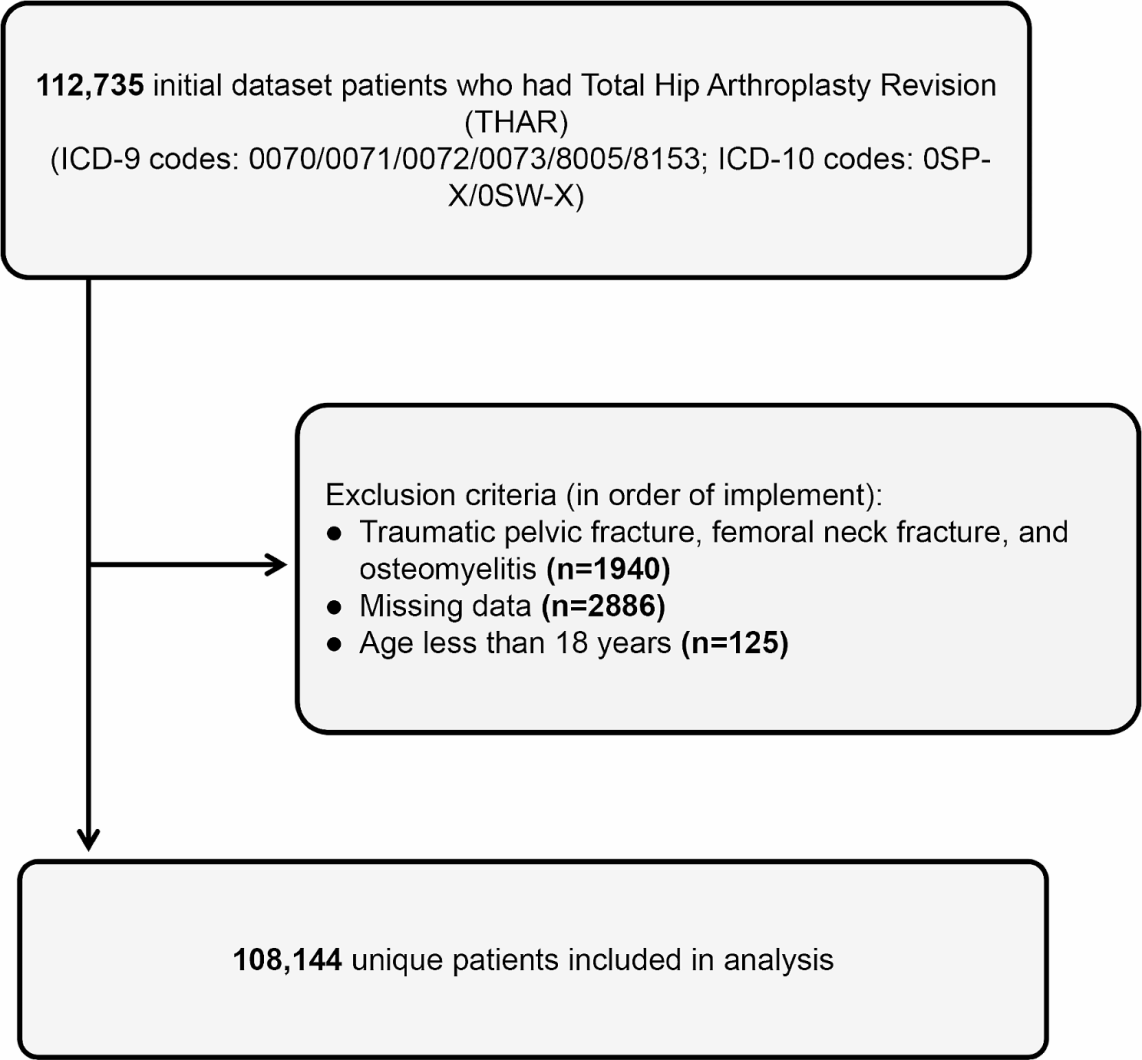
Flow and inclusion/exclusion of all patients had THAR

Exclusion was determined based on the following criteria: (1) patients with traumatic pelvic fracture, femoral neck fracture, and osteomyelitis; (2) data missing; (3) age < 18 years old. In addition, patients with missing race column were defined as other races (Fig. 1).

Patients were divided into two groups according to the presence of PPCs, and those with PPCs were further divided into pneumonia, ARF, and PE groups. Patient demographics including ethnicity, age, and gender were judged. Outcome measures such as the total hospital stay cost, LOS type of insurance, preoperative comorbidities, and postoperative complications were evaluated. The ICD-9-CM and ICD-10-CM diagnostic codes were used to obtain medical and surgical complications prior to discharge.

### Data analyses

R version 4.3.2, a statistical software, was applied to conduct all statistical analyses. The Wilcoxon rank test (continuous data) and the Chi-square test (classified data) were used to assess whether there were significant distinctions between the two study cohorts. In order to explore independent risk factors related to PPCs, multivariate logistic regression was performed using a stepwise regression method. The regression analysis employed all variables simultaneously, including demographics, hospital characteristics, comorbidities, and complications (Table 1). Considering the clinical heterogeneity of PPCs, we investigated risk factors for pneumonia, ARF, and PE separately, utilizing variables identified as risk factors associated with PPCs. Due to the use of a substantial sample size in previous research on NIS, our statistical significance of the alpha level was established to be *P* ≤ 0.001 [8, 10, 11].

**Table 1:** Variables used in binary logistic regression analysis

Variables Categories	Specific Variables
Patient demographics	Age (≤ 64 years and ≥ 65 years), sex (male and female), race (White, Black, Hispanic, Asian or Pacific Islander, Native American and Other)
Hospital characteristics	Type of admission (non-elective, elective), bed size of hospital (small, medium, large), teaching status of hospital (non-teaching, teaching), location of hospital (rural, urban), type of insurance (Medicare, Medicaid, private insurance, self-pay, no charge, other), location of the hospital (northeast, Midwest or north central, south, west)
Comorbidities	AIDS, alcohol abuse, deficiency anemia, rheumatoid diseases, chronic blood loss anemia, congestive heart failure, chronic pulmonary disease, coagulopathy, depression, diabetes (uncomplicated), diabetes (with chronic complications), drug abuse, hypertension, hypothyroidism, liver disease, lymphoma, fluid and electrolyte disorders, metastatic cancer, neurological disorders, obesity, paralysis, peripheral vascular disorders, psychoses, pulmonary circulation disorders, renal failure, solid tumor without metastasis, peptic ulcer disease, valvular disease and weight loss

AIDS: Acquired immunodeficiency syndrome

## Results

### Incidence of PPCs in THAR patients

**Fig. 2:**
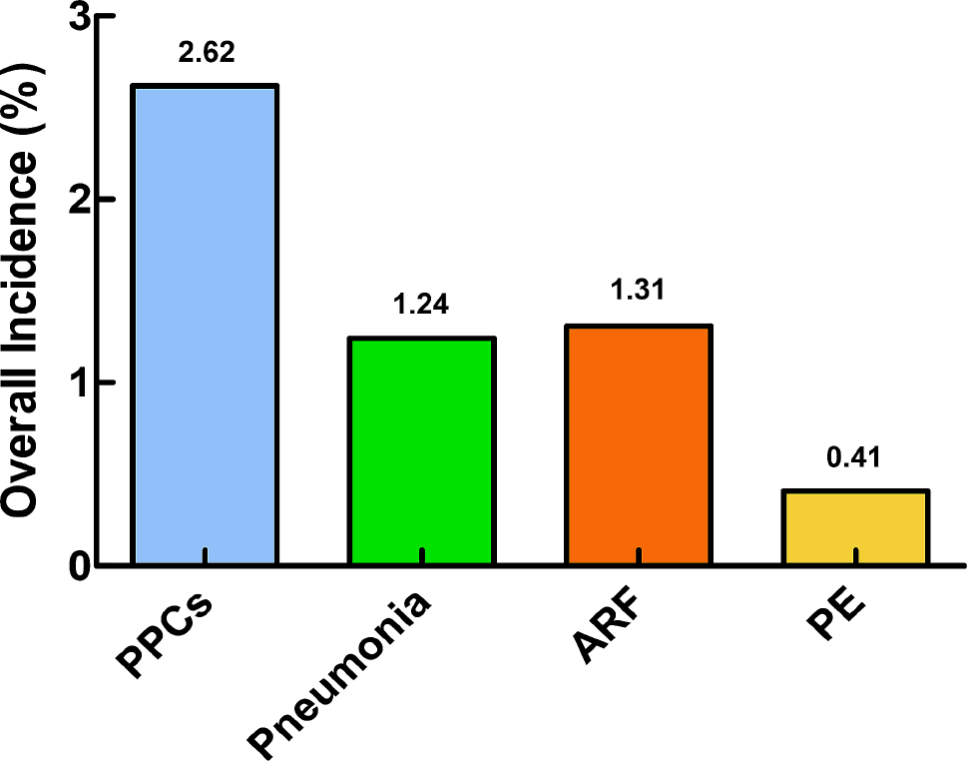
Overall incidences of PPCs and SPCs (pneumonia, ARF, and PE) in patients undergoing THAR

**Fig. 3:**
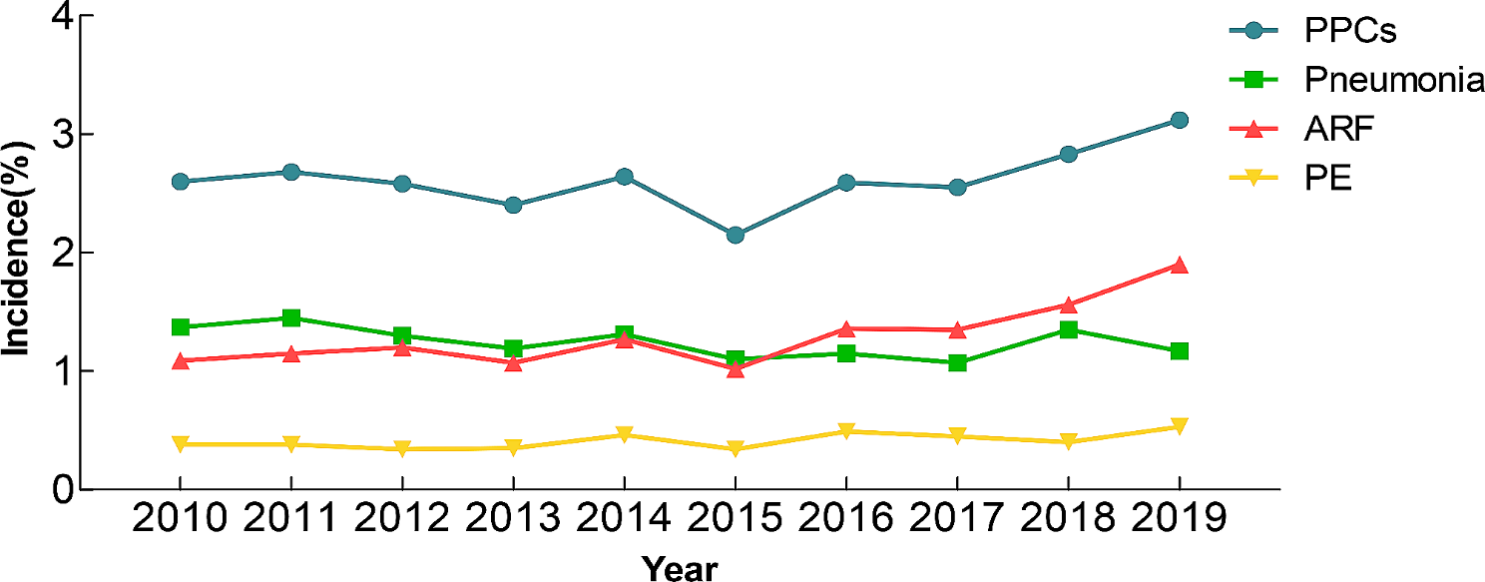
Annual incidences of PPCs and SPCs (pneumonia, ARF, and PE) in patients undergoing THAR

During the period spanning from 2010 to 2019, a comprehensive analysis of the NIS database revealed a total of 112,735 THARs. According to our exclusion criteria, 108,144 patients were included in the analysis (Fig. 1). Across this cohort, PPCs were observed in 2838 patients, with an incidence of 2.62%, including 1346 patients with pneumonia (Overall incidence: 1.24%), 1416 patients with ARF (Overall incidence: 1.31%), 447 patients with PE (Overall incidence: 0.41%) (Fig. 2). Figure 1 presents the incidence of PPCs, pneumonia, ARF, and PE over the years (Fig. 3).

### Patient demographics between the two surgical groups

The age of patients diagnosed with PPCs was found to be, on average, 7 years higher compared to those without PPCs (67 years vs. 74 years, *P* < 0.0001). As expected, there was a notable disparity in the age distribution across the two cohorts, with a much greater prevalence seen in those aged 75 years and beyond (*P* < 0.0001) (Table 2). Nevertheless, there was no discernible disparity in terms of race or gender across the two cohorts within the defined parameters.

**Table 2:** Patient characteristics and outcomes of PPCs after THAR (2010–2019)

Characteristics	No PPCs	PPCs	*P*
Total (n = count)	105,306	2838	
Total incidence (%)	2.62%		
Age (median, years)	67 (58–77)	74 (64–82)	< 0.0001
Age group (%)			
18–44	4.46%	2.68%	< 0.0001
45–64	36.53%	23.75%	
65–74	28.82%	25.26%	
≥ 75	30.19%	48.31%	
Gender (%)			
Male	43.16%	45.84%	0.0044
Female	56.82%	54.16%	
Race (%)			
White	80.74%	81.11%	0.0956
Black	7.05%	6.98%	
Hispanic	3.46%	3.59%	
Asian or Pacific Islander	0.70%	0.67%	
Native American	0.36%	0.28%	
Other	7.70%	7.36%	
Number of Comorbidity (%)			
0	12.78%	1.76%	< 0.0001
1	22.22%	7.26%	
2	23.81%	13.07%	
≥ 3	41.20%	77.91%	
LOS (median, d)	3 (2–6)	9 (6–16)	< 0.0001
TOTCHG (median, $)	70,000 (46,165 − 107,051)	136,095 (81,439 − 222,998)	< 0.0001
Type of insure (%)			
Medicare	63.63%	77.87%	< 0.0001
Medicaid	5.38%	5.88%	
Private insurance	27.40%	13.67%	
Self-pay	0.79%	0.78%	
No charge	0.11%	0.07%	
Other	2.69%	1.73%	
Bed size of hospital (%)			
Small	21.15%	16.46%	< 0.0001
Medium	25.28%	25.19%	
Large	53.57%	58.35%	
Elective admission (%)	69.01%	31.29%	< 0.0001
Type of hospital (teaching, %)	66.58%	66.00%	0.5172
Location of hospital (urban, %)	6.03%	7.19%	0.1065
Died (%)	0.35%	11.42%	< 0.0001
Region of hospital (%)			
Northeast	18.29%	17.62%	0.6314
Midwest or North Central	24.56%	24.56%	
South	37.52%	38.58%	
West	19.62%	19.24%	

PPCs: Postoperative pulmonary complications,

THA: Total hip arthroplasty,

LOS: Length of stay,

TOTCHE: Total charge

### Hospital characteristics between the two surgical groups

As expected, in comparison with patients without PPCs, patients who developed PPCs after THAR were 37.72% less likely to be admitted to hospital through elective admissions (31.29% vs. 69.01%, *P* < 0.0001) (Table 2). Additionally, there was a notable disparity in insurance types between the two groups (*P* < 0.0001), along with a significant difference in the hospital bed size (*P* < 0.0001) (Table 2). However, the disparities in teaching status, location, region, and hospital type between the two cohorts were no statistical significance in our study (Table 2).

### Adverse effects of PPCs after THAR

Overall, patients who developed PPCs after THAR had a 3-fold longer hospital stay, more than 30-fold higher mortality, and 94.4% higher costs compared to those without PPCs (*P* < 0.0001) (Table 2). Moreover, a notable discrepancy was seen in the number of comorbidities across the two groups, with a markedly elevated occurrence among individuals afflicted with three or more comorbidities (*P* < 0.0001) (Table 2).

### Risk and protective factors associated with PPCs after THAR

**Fig. 4:**
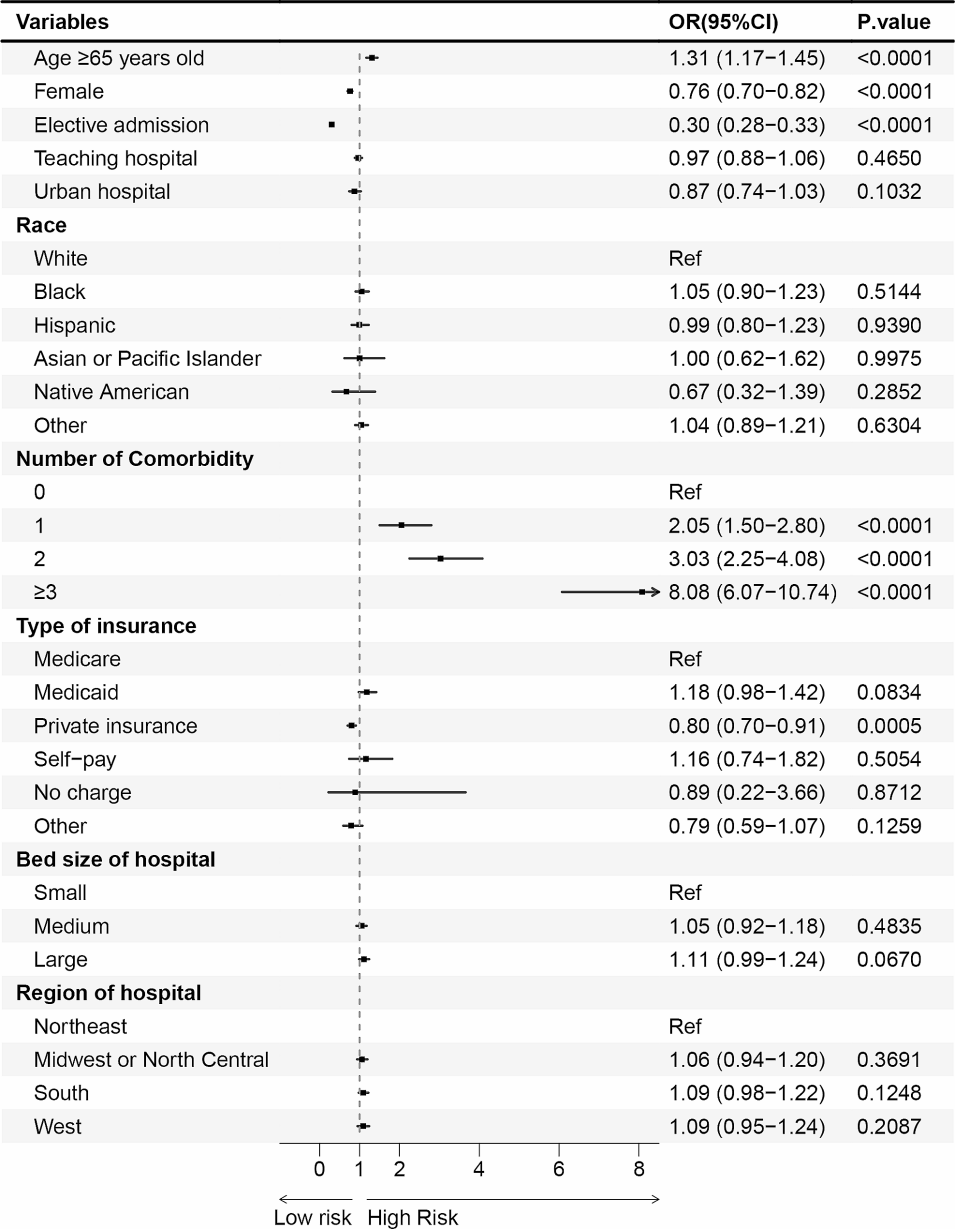
Risk factors associated with PPCs after THAR

Applying logistic regression analysis, we identified the following risk variables in relation to PPCs : advanced age (≥ 65 years; odds ratio [OR] = 1.31; 95% confidence interval [CI] = 1.17–1.45), multiple comorbidities (*n* ≥ 3; OR = 8.08; CI = 6.07–10.74). In addition, 3 protective factors of PPCs including private insurance (OR = 0.80; CI = 0.70–0.91), female (OR = 0.76; CI = 0.70–0.82) and elective admission (OR = 0.30; CI = 0.28–0.33) were observed in our study (Fig. 4).

### Preoperative comorbidities associated with PPCs after THAR

**Fig. 5:**
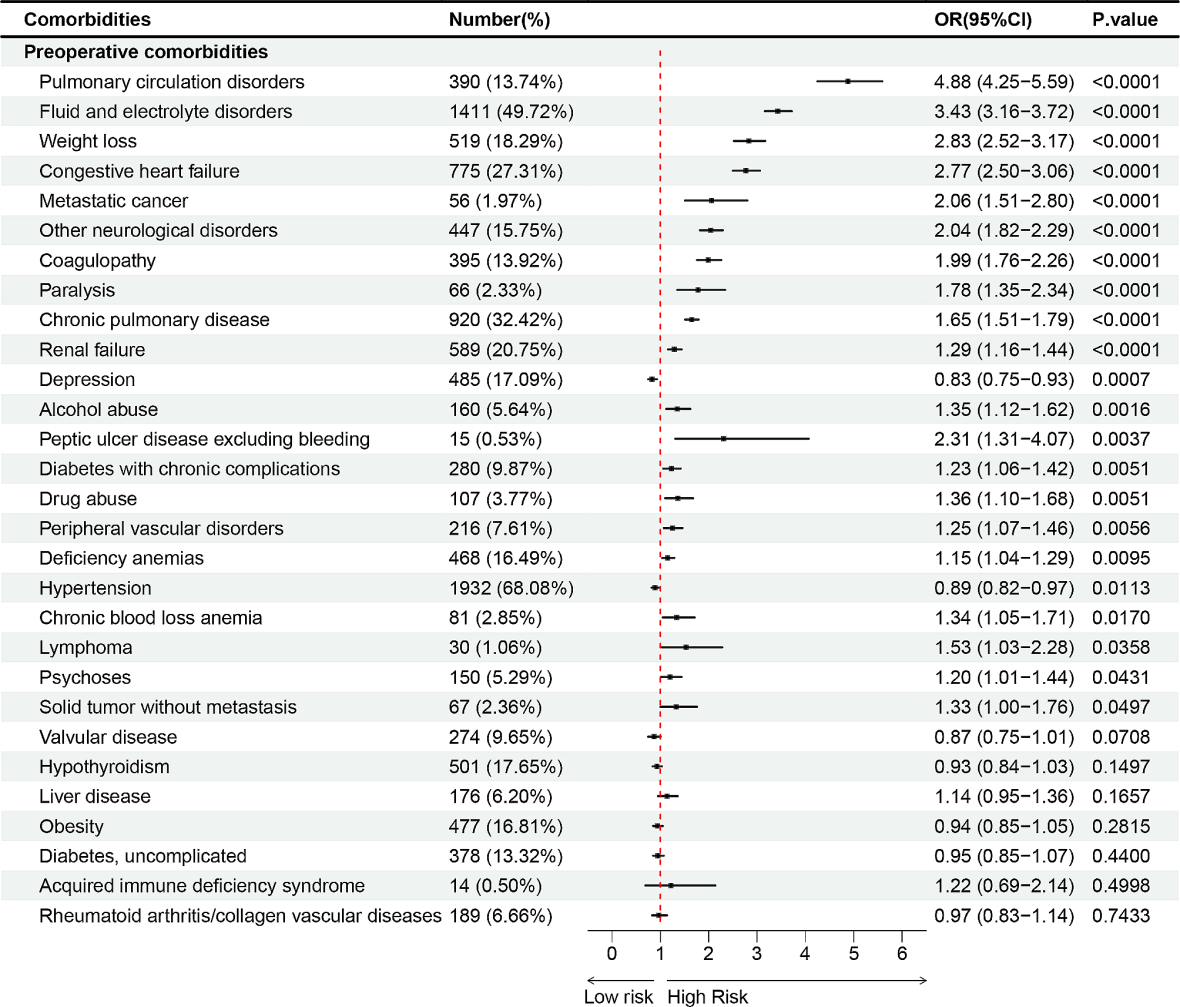
Preoperative comorbidities associated with PPCs after THAR

The preceding analysis indicates that the presence of comorbidities is a significant contributor to the adverse prognosis observed in patients undergoing THAR. Univariate analysis revealed that pulmonary circulation disorders (13.74%), fluid and electrolyte disorders (49.72%), weight loss (18.29%), congestive heart failure (27.31%), metastatic cancer (1.97%), other neurological disorders (15.75%), coagulopathy (13.92%), paralysis (2.33%), chronic pulmonary disease (32.42%), renal failure (20.75%), alcohol abuse (5.64%), peptic ulcer disease excluding bleeding (0.53%), diabetes with chronic complications (9.87%), drug abuse (3.77%), peripheral vascular disorders (7.61%), deficiency anemias (16.49%), hypertension (68.08%), chronic blood loss anemia (2.85%), lymphoma (1.06%), psychoses (5.29%), solid tumor without metastasis (2.36%), valvular disease (9.65%), and liver disease (6.20%) accounted for a higher proportion of patients with PPCs (Table S1). Multivariate Logistic Regression identified that pulmonary circulation disorders (OR = 4.88; CI = 4.25–5.59), fluid and electrolyte disorders (OR = 3.43; CI = 3.16–3.72), weight loss (OR = 2.83; CI = 2.52–3.17), congestive heart failure (OR = 2.77; CI = 2.50–3.06), metastatic cancer (OR = 2.06; CI = 1.51–2.80), other neurological disorders (OR = 2.04; CI = 1.82–2.29), coagulopathy (OR = 1.99; CI = 1.76-2.26), paralysis (OR = 1.78; CI = 1.35–2.34), chronic pulmonary disease (OR = 1.65; CI = 1.51-1.79), renal failure (OR = 1.29; CI = 1.16–1.44) were risk factors for PPCs (Fig. 5).

### Postoperative complications associated with PPCs after THAR

**Fig. 6:**
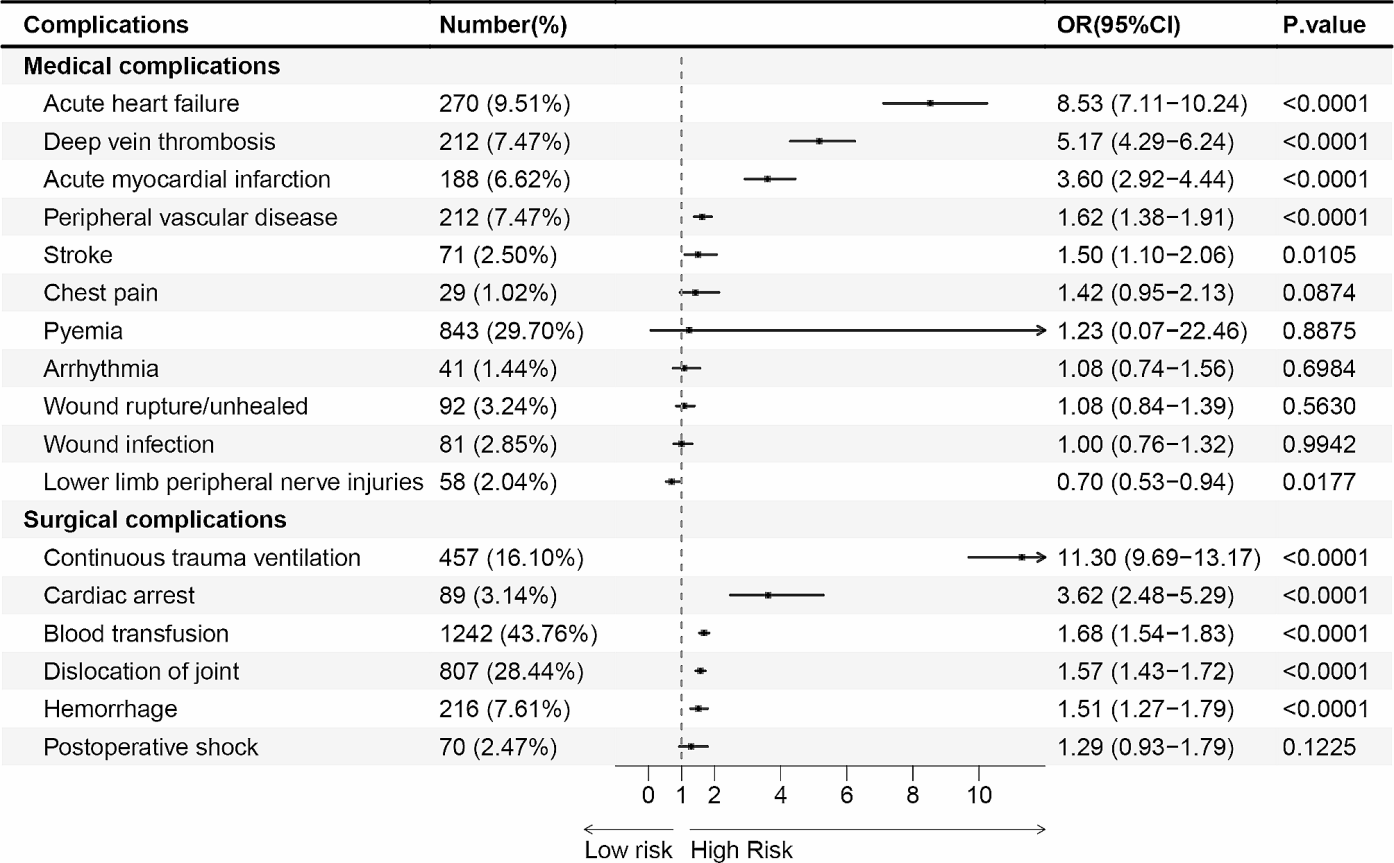
Postoperative complications associated with PPCs after THAR

Postoperative complications emerged as a critical factor in the prognosis of patients undergoing THAR. According to univariate analysis, patients with PPCs were more likely to develop deep vein thrombosis (DVT) (7.47%), cardiac arrest (3.14%), acute myocardial infarction (AMI) (6.62%), acute heart failure (9.51%), peripheral vascular disease (7.47%), blood transfusion (43.76%), postoperative shock (2.47%), stroke (2.50%), arrhythmia (1.44%), continuous trauma ventilation (16.10%), dislocation of joint (28.44%), hemorrhage (7.61%), wound rupture/unhealed (3.24%), wound infection (2.85%), chest pain (1.02%), and pyemia (29.70%) (Table S2). Multivariate analysis presented that PPCs subsequent to THAR were shown to have a significant independent association with acute heart failure (OR = 8.53; CI = 7.11–10.24), deep vein thrombosis (OR = 5.17; CI = 4.29–6.24), acute myocardial infarction (OR = 3.60; CI = 2.92–4.44), peripheral vascular disease (OR = 1.62; CI = 1.38–1.91), stroke (OR = 1.50; CI = 1.10–2.06), continuous trauma ventilation (OR = 11.30; CI = 9.69–13.17), cardiac arrest (OR = 3.62; CI = 2.48–5.29), blood transfusion (OR = 1.68; CI = 1.54–1.83), dislocation of joint (OR = 1.57; CI = 1.43–1.72), hemorrhage (OR = 1.51; CI = 1.27–1.79) (Fig. 6).

### Risk factors associated with SPCs after THAR

**Fig. 7:**
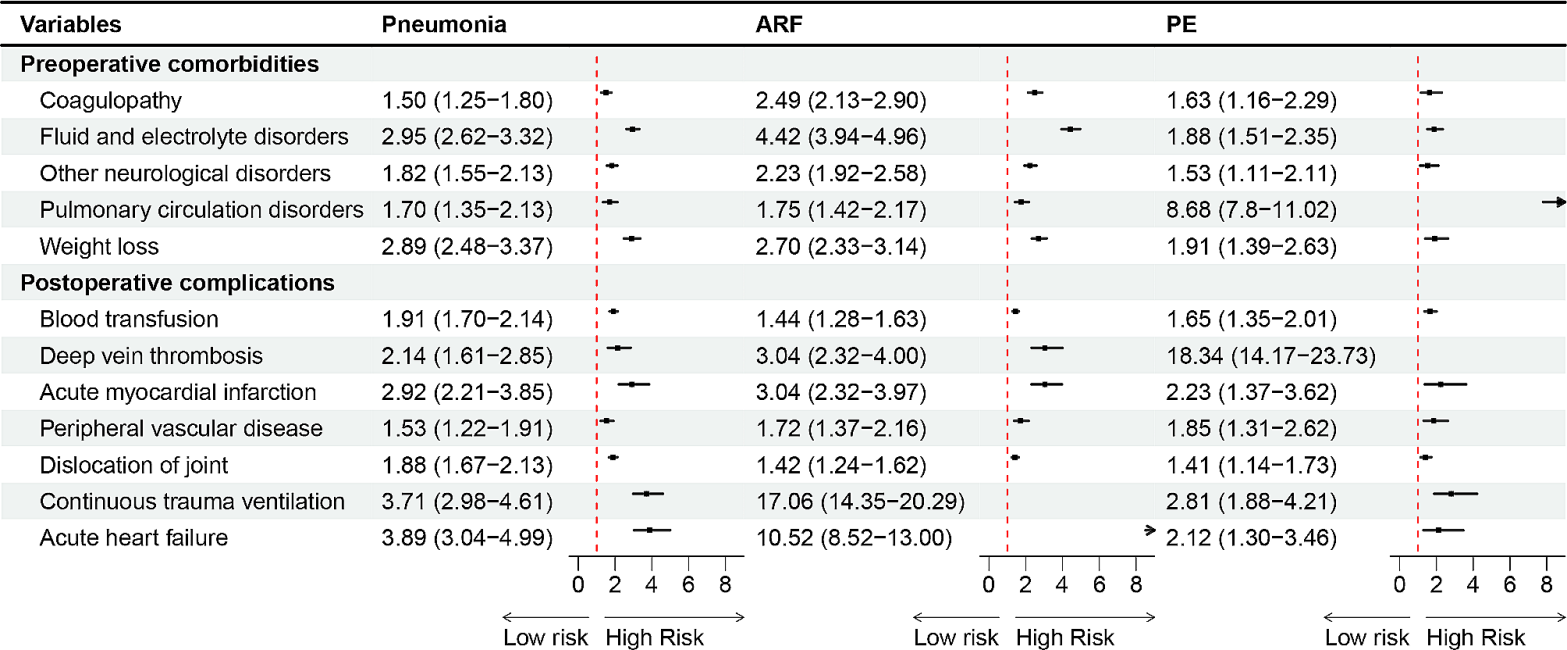
Common risk factors for SPCs (pneumonia, ARF, and PE) after THAR

The risk factors associated with SPCs were presented in Fig. S1. Upon screening, it was identified that coagulopathy, fluid and electrolyte disorders, other neurological disorders, pulmonary circulation disorders, weight loss, postoperative complications, blood transfusions, DVT, AMI, peripheral vascular disease, dislocation of joint, continuous trauma ventilation, and acute heart failure were common risk factors among three specific pulmonary complications (Fig. 7).

## Discussion

This research includes a comprehensive examination of PPCs after THAR, with a focus on the associated health-economic implications. Our research conducted the first national-scale investigation into the incidence of PPCs after THAR at the national level. The incidence of PPCs fluctuated between 2.40% and 2.70% from 2010 to 2014. The lowest rate was observed in 2015, after which there was an increase in the incidence, with the highest incidence in 2019 (3.12%). The change in ICD codes may explain the lower incidence of PPCs in 2015. After October 2015, ICD-CM-10 replaced ICD-CM-9, which may result in missing records of patients with PPCs in some institutions [12]. From 2016 to 2019, the incidence of PPCs and ARF increased simultaneously. One possible explanation is that ICD-CM-10 further subdivides ARF into “ARF with hypoxia” (J9601) and “ARF with hypercapnia” (J9602), implying a more definitive diagnosis of ARF, which may result in the increasing number of patients with ARF and PPCs after 2015.

A previous study of PPCs following THA based on the NIS database found that the incidence of PPCs was no more than 2% between 2004 and 2014 [10]. In comparison with THA patients, THAR patients had a higher incidence of PPCs, which may be attributed to the complexity of THAR surgery in dealing with surgical approach, prosthesis fracture and other issues [13, 14]. This complexity will result in a prolonged procedure time which is a main cause to PPCs [14].

In terms of demographic characteristics, patients with PPCs exhibited a statistically significant age difference of 7 years compared to those without the condition. Additionally, from the age distribution, a clinical observation revealed that the PPCs group had a higher proportion of older patients. Moreover, logistic regression analysis identified an independent risk factor for PPCs as being over 65 years old. Undeniably, numerous studies have consistently demonstrated that advanced age is widely acknowledged as a predictive factor for PPCs [15, 16]. A solid explanation is that several changes occurring with age in the respiratory system, such as chest wall compliance progressively decline and diminished ventilatory response to hypoxia and hypercapnia, can make elderly individuals more susceptible to PPCs [4].

The number of comorbidities for patients with PPCs was significantly higher. This finding is acceptable, as a higher prevalence of comorbidities signifies a comparatively diminished state of health prior to surgery, which could elevate the likelihood of experiencing postoperative consequences, including PPCs. Previous studies have reported that PPCs exhibit a correlation with extended durations of hospitalization, escalated healthcare expenditures, and elevated rates of death [9, 17]. Our study yielded similar results, showing that due to PPCs, the median hospital stay was extended by 6 days, the mortality rate was over 30-fold higher, as well as the overall hospitalization cost per admission rose by $66,095.

Several studies of PPCs have indicated that preoperative screening, appropriate management, and risk stratification are crucial for improving outcomes [4, 6, 18]. To effectively reduce and mitigate the incidence of PPCs after THAR surgery, it is essential to identify individuals with elevated risk prior to the operation [4–6, 19, 20].

Notably, we found that circulatory system diseases were important risk factors for PPCs, including pulmonary circulation disorders (OR = 4.88), DVT (OR = 5.17), and coagulopathy (OR = 1.99). Joyce J et al. have pointed out that coagulopathy due to surgical trauma is a non-negligible risk factor for complications after THAR, leading to an increase in complications such as DVT, pneumonia, and AMI [21]. Previous studies have identified DVT as a trigger for the development of PE [22]. The shedding and metastasis of the thrombus will directly lead to the occurrence of PE and affect other tissues in the lung, causing other PPCs. Pulmonary circulation is regarded as a highly dynamic system. Pulmonary circulation disorders will greatly increase the risk of insufficient blood supply to the lung and hypoxia, resulting in serious surgical outcomes [23].

Cardiac-related complications are one of the significant challenges that cannot be overlooked after THAR surgery. In our study, congestive heart failure (OR = 2.77) was identified as one of the preoperative risk factors for PPCs, which is associated with an increased risk of postoperative pneumonia (OR = 2.69) and (OR = 3.62). As a chronic disease, congestive heart failure has the risk of deterioration into acute heart failure under the action of risk factors such as age and surgery [24]. acute heart failure aggravates cardiac congestion and affects cardiac ejection, which leads to pulmonary circulation obstruction, resulting in severe PPCs such as pulmonary edema, pneumonia, and ARF [25]. Similar to acute heart failure, AMI and cardiac arrest are likewise pivotal risk factors correlated with cardiac complications subsequent to THAR. According to previous studies, cardiac arrest during surgical anesthesia, although rare, is associated with extremely high mortality (70%) [26, 27]. In our study, when cardiac arrest occurred, the risks for both ARF and PE were more than 4-fold higher, which illustrated the importance of guarding against PPCs after cardiac arrest.

Likewise, the contribution of neurological disorders to the development of PPCs in THAR patients merited close consideration. In our study, we identified other neurological disorders as a risk factor for PPCs (OR = 2.04). Neurological system disorders were closely associated with cardiovascular complications, commonly manifesting as heart failure, respiratory failure, and metabolic disorders [28]. Concurrent with cardiovascular dysregulation, patients’ pulmonary function is inevitably compromised. Additionally, previous studies have reported the relationship between intraoperative anesthesia and neurological disorders [29]. Consequently, controlling anesthesia time and optimizing anesthesia strategy were essential ways to reduce the incidence of PPCs in THAR patients [30].

Among the variables examined in our study, continuous trauma ventilation emerged as the most potent predictor for the development of PPCs after THAR (OR = 11.30). Previous studies have reported several ventilator-associated pulmonary events, including pneumonia, ARF, and PE [31, 32]. Notably, an intimate association between continuous trauma ventilation and ARF (OR = 17.06) was observed in our study, indicating an impairment in patients’ respiratory function when utilizing trauma ventilation. Therefore, non-invasive ventilation is one of the critical ways to reduce PPCs during surgery [31].

Matthew Sloan et al. reported a significantly elevated risk of PE in patients with metastatic cancer (OR = 3.07), such a risk factor was identified in our study (OR = 3.69) [33]. In addition, we observed that blood transfusion was associated with an increased risk of developing PPCs (OR = 1.62), especially pneumonia (OR = 1.91) and PE (OR = 1.44). One plausible explanation is that blood transfusion may indicate a disturbance of the patient’s circulatory system during the perioperative period, such as anemia, hypotension and even heart failure [34–36]. Patients with these circulatory disorders may face a higher risk of developing PPCs after THAR surgery.

Previous research has demonstrated a significant association between fluid-electrolyte abnormalities and PPCs [10], which was observed in our study (OR = 3.43). Similarly, as one of the manifestations of fluid imbalance, weight loss is an important risk factor for PPCs (OR = 2.83). It is suggested that fluid-electrolyte imbalances and weight loss may serve as indicators of underlying chronic diseases such as diabetic nephropathy, chronic gastritis, or hypertensive renal damage [10, 37]. Given these findings, patients’ fluid-electrolyte status and weight changes should be closely monitored and managed, thereby optimizing patient outcomes after THAR [37].

There are inherent limitations associated with using NIS databases that should be acknowledged: firstly, it is a retrospective assessment conducted on a national database, which makes it challenging in the examination of particular patient features and factors that influence treatment decision-making; secondly, due to the exclusive reliance on medical billing data in the NIS database, it is possible that coding inconsistencies exist, which could compromise the robustness of multivariate models in accurately capturing observed trends in the development of complications; thirdly, other known risk factors associated with PPCs such as skin moisture, immobility, level of serum albumin, BMI and duration of surgery, are not available within the NIS database [4, 6, 19]. However, the utilization of such an extensive and representative national database like the NIS remains advantageous for identifying trends and demographics due to its widespread use as a reliable research tool [8, 10]. The validity of the NIS database has been confirmed through validation with other databases for accurately identifying patients undergoing specific orthopedic surgeries [8].

## Conclusions

In our study, the aggregate incidence of PPCs was noted at 2.62%, comprising pneumonia at an incidence of 1.24%, ARF at 1.31%, and PE at 0.41%. A range of factors were identified as risk factors for PPCs, including advanced age, pulmonary circulation disorders, fluid and electrolyte disorders, weight loss, congestive heart failure, metastatic cancer, other neurological disorders, coagulopathy, paralysis, chronic pulmonary disease, renal failure, acute heart failure, deep vein thrombosis, acute myocardial infarction, peripheral vascular disease, stroke, continuous trauma ventilation, cardiac arrest, blood transfusion, dislocation of joint, and hemorrhage. Based on these factors, preoperative optimization and management should be prioritized in order to effectively prevent the development of PPCs and improve the postoperative quality of life of patients.

### Electronic supplementary material

Below is the link to the electronic supplementary material.

Supplementary Material 1

Supplementary Material 2

## Data Availability

No datasets were generated or analysed during the current study.

## Abbreviations

THAR: Total hip arthroplasty revision
PPCs: Postoperative pulmonary complications
SPCs: Specific pulmonary complications
ARF: Acute respiratory failure
PE: Pulmonary embolism
DVT: Deep vein thrombosis
AMI: Acute myocardial infarction
NIS: Nationwide Inpatient Sample
LOS: Length of stay
OR: Odds ratio
95% CI: 95% confidence interval
CCI: Charlson comorbidity index
