# Supplementary material for: Incidence and risk factors of postoperative pulmonary complications following total hip arthroplasty revision: a retrospective Nationwide Inpatient Sample database study
Source: J Orthop Surg Res. 2024 Jun 14;19:353. doi: 10.1186/s13018-024-04836-3 (PMC11177359; doi:10.1186/s13018-024-04836-3)
Supplement: Supplementary file 1 — Supplementary Material 1 [file 13018_2024_4836_MOESM1_ESM.docx]

**Table S1** Relationship between PPCs and preoperative comorbidities

| Comorbidities | | Univariate Analysis | | |
| --- | --- | --- | --- | --- |
|  |  | **No** **PPCs** | **PPCs** | **P** |
| Preoperative comorbidities | |  |  |  |
|  | Pulmonary circulation disorders | 1639 (1.56%) | 390 (13.74%) | <0.0001 |
|  | Fluid and electrolyte disorders | 15,889 (15.09%) | 1411 (49.72%) | <0.0001 |
|  | Weight loss | 3241 (3.08%) | 519 (18.29%) | <0.0001 |
|  | Congestive heart failure | 6532 (6.20%) | 775 (27.31%) | <0.0001 |
|  | Metastatic cancer | 614 (0.58%) | 56 (1.97%) | <0.0001 |
|  | Other neurological disorders | 5529 (5.25%) | 447 (15.75%) | <0.0001 |
|  | Coagulopathy | 4169 (3.96%) | 395 (13.92%) | <0.0001 |
|  | Paralysis | 839 (0.80%) | 66 (2.33%) | <0.0001 |
|  | Chronic pulmonary disease | 19,006 (18.05%) | 920 (32.42%) | <0.0001 |
|  | Renal failure | 9172 (8.71%) | 589 (20.75%) | <0.0001 |
|  | Depression | 18,729 (17.79%) | 485 (17.09%) | 0.3386 |
|  | Alcohol abuse | 2761 (2.62%) | 160 (5.64%) | <0.0001 |
|  | Peptic ulcer disease excluding bleeding | 192 (0.18%) | 15 (0.53%) | <0.0001 |
|  | Diabetes with chronic complications | 4770 (4.53%) | 280 (9.87%) | <0.0001 |
|  | Drug abuse | 2194 (2.08%) | 107 (3.77%) | <0.0001 |
|  | Peripheral vascular disorders | 3678 (3.49%) | 216 (7.61%) | <0.0001 |
|  | Deficiency anemias | 10,872 (10.32%) | 468 (16.49%) | <0.0001 |
|  | Lymphoma | 524 (0.50%) | 30 (1.06%) | <0.0001 |
|  | Psychoses | 3652 (3.47%) | 150 (5.29%) | <0.0001 |
|  | Solid tumor without metastasis | 997 (0.95%) | 67 (2.36%) | <0.0001 |
|  | Valvular disease | 5105 (4.85%) | 274 (9.65%) | <0.0001 |
|  | Hypothyroidism | 17,528 (16.64%) | 501 (17.65%) | 0.1549 |
|  | Liver disease | 3005 (2.85%) | 176 (6.20%) | <0.0001 |
|  | Obesity | 18,323 (17.40%) | 477 (16.81%) | 0.4114 |
|  | Diabetes, uncomplicated | 14,145 (13.43%) | 378 (13.32%) | 0.8616 |
|  | Acquired immune deficiency syndrome | 354 (0.34%) | 14 (0.49%) | 0.1560 |
|  | Rheumatoid arthritis/collagen vascular diseases | 6882 (6.54%) | 189 (6.66%) | 0.7914 |

OR: Odd ratio,

CI: Confidence interval,

PPCs: Postoperative pulmonary complications

**Table S2** Relationship between PPCs and postoperative complications

| Complications | | Univariate Analysis | | |
| --- | --- | --- | --- | --- |
|  |  | **No PPCs** | **PPCs** | **P** |
| Medical complications | |  |  |  |
|  | Deep vein thrombosis | 850 (0.81%) | 212 (7.47%) | <0.0001 |
|  | Cardiac arrest | 134 (0.13%) | 89 (3.14%) | <0.0001 |
|  | Acute myocardial infarction | 739 (0.70%) | 188 (6.62%) | <0.0001 |
|  | Acute heart failure | 4343 (4.12%) | 270 (9.51%) | <0.0001 |
|  | Blood transfusion | 25,664 (24.37%) | 1242 (43.76%) | <0.0001 |
|  | Peripheral vascular disease | 3605 (3.42%) | 212 (7.47%) | <0.0001 |
|  | Postoperative shock | 415 (0.34%) | 70 (2.47%) | <0.0001 |
|  | Stroke | 817 (0.78%) | 71 (2.50%) | <0.0001 |
|  | Arrhythmia | 894 (0.85%) | 41 (1.44%) | 0.0007 |
| Surgical complications | |  |  |  |
|  | Continuous trauma ventilation | 691 (0.66%) | 457 (16.10%) | <0.0001 |
|  | Dislocation of joint | 25,643 (24.35%) | 807 (28.44%) | <0.0001 |
|  | Hemorrhage | 2889 (2.74%) | 216 (7.61%) | <0.0001 |
|  | Wound rupture/unhealed | 1507 (1.43%) | 92 (3.24%) | <0.0001 |
|  | Wound infection | 1142 (1.08%) | 81 (2.85%) | <0.0001 |
|  | Chest pain | 655 (0.62%) | 29 (1.02%) | 0.0080 |
|  | Pyemia | 2749 (2.61%) | 843 (29.70%) | <0.0001 |
|  | Lower limb peripheral nerve injuries | 2530 (2.40%) | 58 (2.04%) | 0.2171 |

OR: Odd ratio,

CI: Confidence interval,

PPCs: Postoperative pulmonary complications

**Table S3** Definition of Bedsize of hospital in the NIS database

| **Bedsize of hospital** | | | |
| --- | --- | --- | --- |
| **Location and Teaching Status** | **Hospital Bedsize (Number)** | | |
|  | Small | Medium | Large |
| **NORTHEAST REGION** | | | |
| Rural | 1-49 | 50-99 | 100+ |
| Urban, nonteaching | 1-124 | 125-199 | 200+ |
| Urban, teaching | 1-249 | 250-424 | 425+ |
| **MIDWEST REGION** | | | |
| Rural | 1-29 | 30-49 | 50+ |
| Urban, nonteaching | 1-74 | 75-174 | 175+ |
| Urban, teaching | 1-249 | 250-374 | 375+ |
| **SOUTHERN REGION** | | | |
| Rural | 1-39 | 40-74 | 75+ |
| Urban, nonteaching | 1-99 | 100-199 | 200+ |
| Urban, teaching | 1-249 | 250-449 | 450+ |
| **WESTERN REGION** | | | |
| Rural | 1-24 | 25-44 | 45+ |
| Urban, nonteaching | 1-99 | 100-174 | 175+ |
| Urban, teaching | 1-199 | 200-324 | 325+ |

NIS: National Inpatient Sample
