# Supplementary figures and images for: Incidence and risk factors of postoperative pulmonary complications following total hip arthroplasty revision: a retrospective Nationwide Inpatient Sample database study
Source: J Orthop Surg Res. 2024 Jun 14;19:353. doi: 10.1186/s13018-024-04836-3 (PMC11177359; doi:10.1186/s13018-024-04836-3)

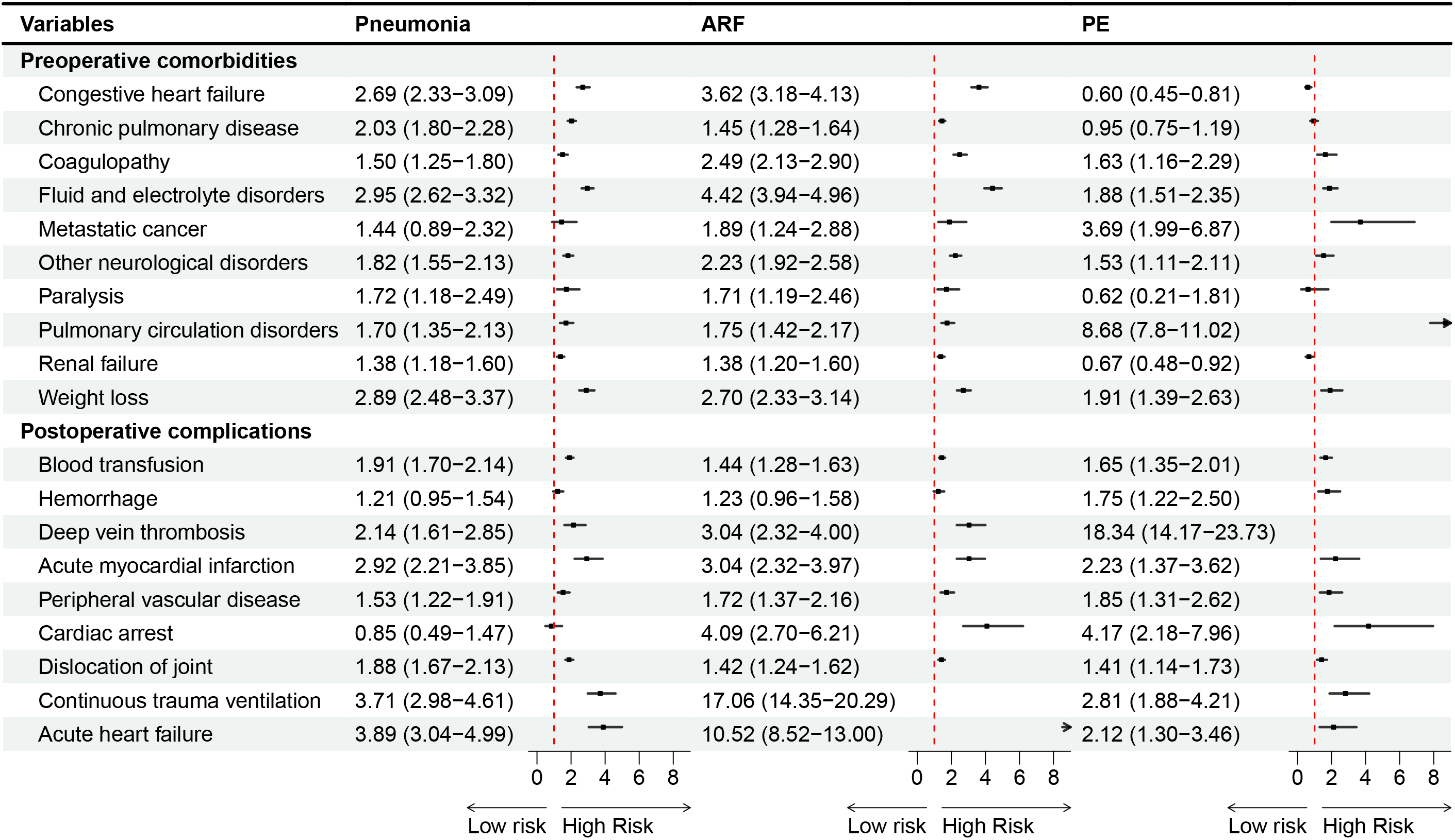

Supplement: Supplementary file 2 — Supplementary Material 2 [file 13018_2024_4836_MOESM2_ESM.tif]
